# Effectiveness and safety of endovascular therapy compared to intravenous thrombolysis in acute ischaemic stroke due to medium-vessel occlusions: a real-world multicentre study from the Italian SITS registry

**DOI:** 10.1093/esj/aakag020

**Published:** 2026-03-29

**Authors:** Matteo Farè, Elisa Bianchi, Giulia Benina, Gabriele Lucchi, Martina Mercenari, Francesco Innocenti, Stefano Spinelli, Giovanni Isgrò, Romano Orofino, Michela Galimberti, Edoardo Pedranzini, Giorgia Orsani, Angela Giglio, Francesco Andrea Pedrazzini, Francesco Pasini, Danilo Antonio Montisano, Francesco Santangelo, Nicola Rifino, Martina Viganò, Claudia Balducci, Riccardo Altavilla, Salvatore Amarù, Bonaventura Ardito, Luigi Bartolomei, Margherita Bellucci, Guido Bigliardi, Giovanni Boero, Roberto Bombardi, Giovanni Bosco, Ivana Bosone, Giuseppina Calabrese, Luigi Caputi, Angelo Cascio Rizzo, Roberto Cavallo, Alberto Chiti, Elena Coco, Letizia Concari, Paolo Del Dotto, Massimo Del Sette, Maria Luisa Dell'Acqua, Delfina Ferrandi, Massimo Filippi, Stefano Forlivesi, Mauro Galletti, Antonio Gasparro, Mauro Gentile, Laura Godi, Paolo Invernizzi, Nicola Iorio, Patrizia Julita, Marco Longoni, Vincenzo Lucivero, Mauro Magoni, Fabio Marchioretto, Alessandra Martignoni, Emanuele Medici, Valerio Melas, Stefano Meletti, Fabio Melis, Maurizio Melis, Gabriella Monteforte, Ciro Mundi, Stefano Novello, Cristina Paci, Carmela Palmieri, Matteo Paolucci, Maria Giovanna Pennisi, Silvio Piffer, Enza Pinto, Maurizio Plocco, Maurizia Rasura, Giuseppe Rinaldi, Pier Andrea Rizzo, Marco Vito Rossi, Luisa Roveri, Fabrizio Sallustio, Andrea Salmaggi, Alessandra Sanna, Mariantonietta Savarese, Maria Sessa, Emanuele Spina, Silvia Strumia, Roberto Tarletti, Tiziana Tassinari, Giuseppe Torgano, Alessandro Trebbastoni, Laura Vandelli, Marco Vista, Andrea Zini, Danilo Toni, for the SITS-MeVO Study Group, Agostoni Elio, Baldi Antonio, Cardinali Patrizio, Cavallini Anna, Ferrarese Carlo, Guidotti Mario, Martusciello Gioacchino, Meineri Piero, Napoletano Rosa, Pasquinucci Antonella, Plutino Andrea, Reia Antonio, Scaglione Gaspare, Sicurella Luigi, Tassi Rossana, Simone Beretta

**Affiliations:** Department of Neurology, Fondazione IRCCS San Gerardo dei Tintori, University of Milano Bicocca, Monza, Italy; Laboratorio di Neurologia, Istituto di Ricerche Farmacologiche Mario Negri IRCCS, Milan, Italy; Department of Neurology, Fondazione IRCCS San Gerardo dei Tintori, University of Milano Bicocca, Monza, Italy; Department of Neurology, Fondazione IRCCS San Gerardo dei Tintori, University of Milano Bicocca, Monza, Italy; Department of Neurology, Fondazione IRCCS San Gerardo dei Tintori, University of Milano Bicocca, Monza, Italy; Department of Neurology, Fondazione IRCCS San Gerardo dei Tintori, University of Milano Bicocca, Monza, Italy; Department of Neurology, Fondazione IRCCS San Gerardo dei Tintori, University of Milano Bicocca, Monza, Italy; Department of Neurology, Fondazione IRCCS San Gerardo dei Tintori, University of Milano Bicocca, Monza, Italy; Department of Neurology, Fondazione IRCCS San Gerardo dei Tintori, University of Milano Bicocca, Monza, Italy; Department of Neurology, Fondazione IRCCS San Gerardo dei Tintori, University of Milano Bicocca, Monza, Italy; Department of Neurology, Fondazione IRCCS San Gerardo dei Tintori, University of Milano Bicocca, Monza, Italy; Department of Neurology, Fondazione IRCCS San Gerardo dei Tintori, University of Milano Bicocca, Monza, Italy; Department of Neurology, Fondazione IRCCS San Gerardo dei Tintori, University of Milano Bicocca, Monza, Italy; Department of Neurology, Fondazione IRCCS San Gerardo dei Tintori, University of Milano Bicocca, Monza, Italy; Department of Neurology, Fondazione IRCCS San Gerardo dei Tintori, University of Milano Bicocca, Monza, Italy; Department of Neurology, Fondazione IRCCS San Gerardo dei Tintori, University of Milano Bicocca, Monza, Italy; Department of Neurology, Fondazione IRCCS San Gerardo dei Tintori, University of Milano Bicocca, Monza, Italy; Department of Neurology, Fondazione IRCCS San Gerardo dei Tintori, University of Milano Bicocca, Monza, Italy; Department of Neurology, Fondazione IRCCS San Gerardo dei Tintori, University of Milano Bicocca, Monza, Italy; Department of Neurology, Fondazione IRCCS San Gerardo dei Tintori, University of Milano Bicocca, Monza, Italy; Neurology and Stroke Unit, P.O. San Carlo Borromeo, ASST Santi Paolo e Carlo, Milan, Italy; Department of Neurology, Infermi Hospital, Rivoli, Italy; Department of Neurology and Stroke Unit, Della Murgia Fabio Perinei Hospital, Altamura I-70022, Italy; Neurological Department, Mirano Hospital, ULSS3 Serenissima, via don G. Sartor 4, Mirano 30035, Italy; Neurology and Stroke Unit, S. Corona Hospital, Pietra Ligure, Florene, Italy; Department of Neurology, Ospedale Civile di Baggiovara, AOU Modena, Modena, Italy; Department of Neurology, SS Annunziata Hospital, Taranto, Italy; Neurology Unit and Stroke Unit, Ospedale Alto Vicentino, Santorso, Vicenza, Italy; Department of Neuroscience, Stroke Unit, University of Torino, Torino, Italy; Department of Neurology, AO Ordine Mauriziano, Torino, Italy; Department of Neurology, Desio Hospital, Azienda Socio Sanitaria Territoriale della Brianza, Desio, Italy; Neurology Unit, Department of Cardio-Cerebrovascular Diseases, Maggiore Hospital ASST-Crema, Crema, Cremona, Italy; Department of Neurology and Stroke Unit, ASST Grande Ospedale Metropolitano Niguarda, Milan, Italy; Neurology Unit, San Giovanni Bosco Hospital, Turin, Italy; Unit of Neurology, Apuane Hospital, Massa, Massa-Carrara, Italy; Neurology and Stroke Unit, S. Corona Hospital, Pietra Ligure, Florene, Italy; UO Neurologia, Presidio Ospedaliero di Fidenza AUSL, Parma, Fidenza, Italy; Unit of Neurology, Azienda USL Toscana Nord-Ovest, Versilia Hospital, Via Aurelia, 335, Lido di Camaiore, Lucca 55041, Italy; Department of Neuroscience, IRCCS Ospedale Policlinico San Martino, Genoa, Italy; Department of Neurology, Ospedale Civile di Baggiovara, AOU Modena, Modena, Italy; Stroke Unit-Department of Neurology, SS. Biagio e Arrigo Hospital, Alessandria, Italy; Vita-Salute San Raffaele University, Milano, Italy; Neurology Unit, Neurorehabilitation Unit, and Neurophysiology Service, IRCCS San Raffaele Scientific Institute, Milano, Italy; IRCCS Istituto Delle Scienze Neurologiche Di Bologna, Department of Neurology and Stroke Center, Maggiore Hospital, Bologna, Italy; Neurology Unit, Ospedale Santo Spirito, Casale Monferrato, Alessandria, Italy; Department of Neurology and Stroke Unit, A.O.O.R. Villa Sofia-V. Cervello, Palermo, Italy; IRCCS Istituto Delle Scienze Neurologiche Di Bologna, Department of Neurology and Stroke Center, Maggiore Hospital, Bologna, Italy; Department of Neurology, ASL NO, Borgomanero Hospital, Viale Zoppis 10, Borgomanero, Novara 28021, Italy; Neurology Unit, Istituto Ospedaliero Fondazione Poliambulanza, Brescia, Italy; PO Stroke Unit, Ospedale Cardarelli, Campobasso, Italy; Neurology Unit, San Biagio Hospital, Domodossola, Italy; Neurology and Stroke Unit, Department of Neuroscience, Bufalini Hospital, Cesena, Italy; Neurology Unit, Ospedale “Dimiccoli”, Barletta, Italy; Stroke Unit, Azienda Socio Sanitaria Territoriale Spedali Civili, Brescia, Italy; Neurological Unit, IRCCS Sacro Cuore Don Calabria Hospital, Verona, Italy; Unit of Cardiac and Cerebrovascular Disease-General Medicine 2, Foundation I.R.C.C.S. Policlinico San Matteo, University of Pavia, Pavia, Italy; Neurology Unit, Ospedale Civile Macerata, Macerata, Italy; Department of Medical Sciences and Public Health, University of Cagliari, Cagliari, Italy; Department of Neurology, Ospedale Civile di Baggiovara, AOU Modena, Modena, Italy; S.S. NeuroVascolare, Ospedale Maria Vittoria, ASL Città di Torino, Torino, Italy; Neurology Service and Stroke Unit, Department of Neuroscience, AO Brotzu, Cagliari, Italy; Stroke Unit, “Santa Maria Goretti” Hospital, Latina, Italy; Department of Neuroscience, United Hospital of Foggia, Foggia 71100, Italy; Department of Neurology, Azienda Sanitaria Friuli Occidentale, Pordenone, Italy; Neurology Unit, Ospedale Provinciale “Madonna del Soccorso”, San Benedetto del Tronto 63074, Italy; Medical Department, E. Agnelli Hospital—Local Health Company (ASL) TO3, Pinerolo, Italy; IRCCS Istituto Delle Scienze Neurologiche Di Bologna, Department of Neurology and Stroke Center, Maggiore Hospital, Bologna, Italy; Neurology Unit, Cannizzaro Hospital, Catania, Italy; Neurology Unit, Trento Hospital, Azienda Provinciale per i Servizi Sanitari (APSS) di Trento, Trento 38122, Italy; Neurological Department, Antonio Perrino Hospital, Brindisi, Italy; UTN (Neurovascular Therapy Unit), F. Spaziani Hospital, Frosinone, Italy; Stroke Unit, Saint Andrea Hospital, La Sapienza University, Rome, Italy; Department of Neurology, Di Venere Hospital, Bari, Italy; UOC di Neurologia, Fondazione Policlinico Universitario A. Gemelli IRCCS, Roma, Italy; Department of Neurology, Di Venere Hospital, Bari, Italy; Vita-Salute San Raffaele University, Milano, Italy; Stroke Unit, Ospedale dei Castelli, ASL Roma 6, Ariccia, Italy; Department of Neurology and Stroke Unit, ASST Lecco Ospedale Alessandro Manzoni, Lecco, Italy; Stroke Unit, AOU Sassari, Sassari, Italy; “FM Puca” Neurology Unit, University Hospital Consortium Corporation Polyclinic of Bari, Bari 70124, Italy; Department of Neurology, ASST Papa Giovanni XXIII, Bergamo, Italy; Neurology and Stroke Unit, “A. Cardarelli” Hospital, Naples, Italy; Department of Neurology, G.B. Morgagni L. Pierantoni Hospital, Forlì, Italy; SCDU Neurologia—Stroke Unit, Azienda Ospedaliero-Universitaria “Maggiore della Carità”, Novara, Italy; Neurology and Stroke Unit, S. Corona Hospital, Pietra Ligure, Florene, Italy; Emergency Care Unit, Fondazione IRCCS Ca' Granda OspedaleMaggiore Policlinico, Via F. Sforza 35, Milan 20100, Italy; Stroke Unit, Ospedale dei Castelli, ASL Roma 6, Ariccia, Italy; Department of Neurology, Ospedale Civile di Baggiovara, AOU Modena, Modena, Italy; Unit of Neurology, San Luca Hospital, Via Lippi-Francesconi, Lucca 55100, Italy; IRCCS Istituto Delle Scienze Neurologiche Di Bologna, Department of Neurology and Stroke Center, Maggiore Hospital, Bologna, Italy; Emergency Department Stroke Unit, Department of Human Neurosciences, Sapienza University of Rome, Rome, Italy; Department of Neurology, Fondazione IRCCS San Gerardo dei Tintori, University of Milano Bicocca, Monza, Italy

**Keywords:** acute ischaemic stroke, endovascular therapy, intravenous thrombolysis, MeVO, real-world data

## Abstract

**Introduction:**

Recent randomised trials have questioned the benefit of endovascular therapy (EVT) for MeVO stroke, but data from clinical practice are limited. This study aimed to assess the effectiveness and safety of EVT, with or without intravenous thrombolysis (IVT), vs IVT alone in MeVO stroke using registry-based real-world data.

**Patients and methods:**

This retrospective multicentre study included patients from 82 Italian centres in the Safe Implementation of Treatments in Stroke (SITS) registry (January 2020–December 2023). Adults with acute ischaemic stroke due to MeVO (ACA A1/A2, MCA M2/M3 or more distal or PCA P1/P2), treated with IVT or EVT ± IVT, and with available 90-day mRS scores were included. Patients with tandem occlusions were excluded. Propensity score matching (1:1) was used to balance baseline variables. Primary outcome was functional independence (mRS 0–2) at 90 days. Secondary outcomes included in-hospital mortality, intracranial haemorrhage incidence and recanalisation status.

**Results:**

Among 1375 total patients, 780 were included and matched (390 per group) by propensity score. Baseline characteristics were balanced. Functional independence at 90 days was achieved in 60.6% of EVT ± IVT patients vs 60.9% in the IVT-only group (odds ratio [OR] 0.99; 95% CI, 0.73–1.34; *P* = .939). When restricted to patients with baseline mRS < 2, functional independence rates remained comparable between groups, confirming the primary findings. In-hospital mortality was non-significantly lower in the EVT ± IVT group (5.4% vs 8.7%, *P* = .069). Symptomatic intracranial haemorrhage rates were comparable between groups, although overall haemorrhagic complications were higher with EVT (18.4% vs 11.2%, *P* < .0001). Stratified analyses by stroke severity and treatment timing showed consistent lack of benefit across all subgroups (all interaction *P*-values > .05).

**Discussion:**

The absence of functional benefit from EVT observed in this real-world cohort is consistent with the results of the ESCAPE-MeVO and DISTAL randomized trials. Notably, the higher rate of any intracranial haemorrhage in the EVT group (18.4% vs 11.2%), driven primarily by minor haemorrhagic events, represents a clinically meaningful safety concern that must be weighed against the lack of demonstrated efficacy. A hypothesis-generating signal was observed in patients treated within 180 minutes (OR 2.16, 95% CI 1.06–4.38), warranting prospective investigation. The retrospective design and the limitations inherent to registry-based data, including incomplete procedural data and anatomical heterogeneity in MeVO classification, should be considered when interpreting these findings.

**Conclusions:**

Endovascular therapy did not improve long-term functional outcomes compared to IVT alone in MeVO stroke but was associated with higher haemorrhagic risk. These findings support a cautious approach to EVT in this setting, in line with recent trial evidence.

## Graphical Abstract

**Graphical Abstract:**
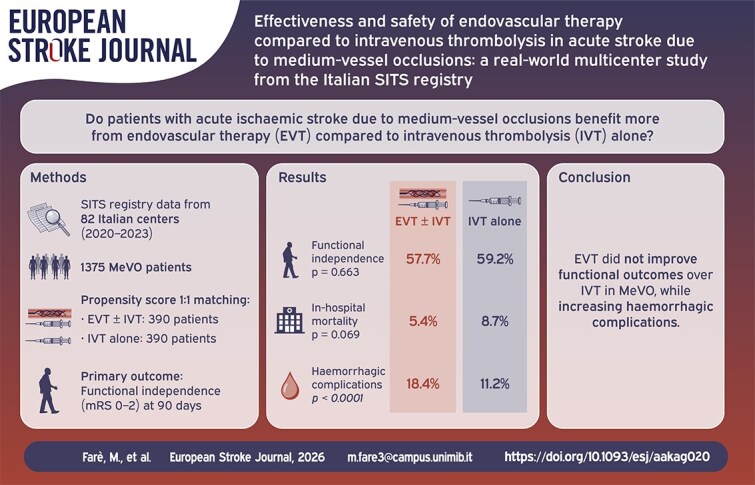

## Introduction

Acute ischaemic stroke is the main neurological disease needing emergency care, especially since revascularisation therapies were introduced.^1^ The highest mortality and disabling outcome of ischaemic stroke is associated with LVOs, such as intracranial internal carotid artery and M1 segment of MCA occlusions.^2^ On the other hand, MeVO usually bears an intrinsic better outcome compared to LVO, since the area of cerebral tissue affected by the hypoperfusion is smaller and can benefit from a larger collateral network.^3^ However, despite the intrinsic better scenario, most patients with ischaemic stroke attributed to MeVO do not reach a satisfactory outcome, with over 30% of them not regaining functional independence at 3 months after the event.^3^

Intravenous thrombolysis (IVT) shows clearer efficacy in MeVO than LVO, given the smaller size of the thrombus, despite an early recanalisation rate not exceeding 50%.^4^ Endovascular mechanical thrombectomy (EVT) provides evidence-based benefit in LVO ischaemic strokes.^5^ However, the utility of EVT in MeVO has been derived from trials which focused on the overall benefit of the procedure, often not taking vessel size into account, and other observational studies did not show significant effectiveness compared to IVT.^6–8^ Two large randomised controlled trials, ESCAPE-MeVO and DISTAL, were published in 2025 and brought new evidence on this topic, both demonstrating no reduction in disability and mortality with EVT compared with best medical treatment, which included IVT if appropriate.^9,10^

This study, Safe Implementation of Treatments in Stroke (SITS)-MeVO, aims to assess the benefit of performing thrombectomy, in addition to standard medical therapy, compared to IVT alone, on MeVO stroke outcomes in a routine care setting, using real-world data from the Italian centres participating in the SITS registry.

## Materials and methods

The study was designed as a retrospective, multicentre, registry-based study, in which we included consecutive patients enrolled between 1st January 2020 and 31st December 2023, who met the following criteria: (1) age ≥ 18 years; (2) acute ischaemic stroke due to MeVO, defined as per registry options as occlusion in anterior cerebral artery (ACA) A1/A2, MCA M2/M3 or more distal or posterior cerebral artery (PCA) P1/P2 segments on CT angiography; (3) treatment with IVT or EVT ± IVT (IVT included only alteplase, as tenecteplase was not available in the study timeframe); (4) absence of tandem occlusions; (5) availability of 90-day mRS score.

The SITS registry categorises M3 and M4 occlusions together under “M3 or more distal” and does not differentiate between dominant and non-dominant M2 branches; therefore, all M2 occlusions were included regardless of dominance pattern, constituting a limitation, as dominant M2 occlusions are often classified as LVOs in other studies. Moreover, tandem lesions were excluded, consistently with ESCAPE-MeVO and DISTAL trials, because they represent a distinct pathophysiological entity with different treatment algorithms and expected outcomes that would confound the comparison between pure MeVO treatment strategies.

The primary outcome was functional independence at 90 days, defined as mRS score 0–2 in the 2 treatment groups, being the most widely validated endpoint in stroke trials, including ESCAPE-MeVO and DISTAL, and facilitating direct comparison with the existing evidence base. Secondary outcomes included in-hospital mortality in the 2 treatment groups and safety endpoints which included in-hospital and overall mortality, occurrence of symptomatic intracranial haemorrhage according to SITS Monitoring Study (SITS-MOST) criteria,^11^ and recanalisation status when available.

From the initial cohort of 1375 patients meeting inclusion criteria, 444 (32.3%) had undergone EVT. Among these, 54 patients were excluded due to missing data on primary outcome or key matching variables, resulting in 390 EVT patients. To minimise confounding and selection bias, treatment groups were compared using a matched cohort design: a propensity score was calculated using a logistic regression model, with treatment (EVT ± IVT vs IVT only) as dependent variable and baseline demographic and clinical variables which were considered as possible confounders. Variables included in the propensity score model were: age, baseline NIHSS score, pre-stroke mRS, vascular risk factors (hypertension, diabetes mellitus, hyperlipidaemia, current smoking, atrial fibrillation, previous stroke), onset-to-treatment time metrics, imaging features (dense artery sign as a marker of thrombus burden^12^), occlusion site and occlusion side. Patients were matched 1:1 by both site of occlusion and propensity score using nearest-neighbour matching without replacement.

The final analyses were conducted on the matched population only. Descriptive statistics on the main demographic and clinical variables were performed in the entire sample and comparing the 2 treatment groups. Categorical variables were reported as counts and percentages, continuous variables as medians with interquartile ranges. Group comparisons were performed using chi-square or Fisher’s exact tests for categorical variables and Wilcoxon–Mann–Whitney test for continuous variables. The odds ratio (OR) for good clinical outcome was estimated using logistic regression models with treatment group as the independent variable and treatment group as dependent variable. An ordinal analysis on functional outcome as measured by the mRS adjusted by the baseline functional status was also performed using a multinomial ordinal logistic regression model, with the mRS ordinal score as dependent variable, treatment as independent variable and baseline mRS score as covariate. A 90-day survival analysis comparing the 2 treatment groups was also performed with Kaplan–Meier survival curves and log-rank test. Stratified analyses of the primary outcome by NIHSS categories (<5, 5–15, > 15) and onset-to-treatment time (≤180 min, > 180 min) were performed, testing first treatment-by-covariate interactions to assess the presence of significant differences in the effect of treatment between strata. Guidelines typically recommend EVT for NIHSS > 5, we included patients with NIHSS < 5 because registry data showed these patients were treated with EVT in real-world practice, and this stratification allows examination of treatment effects across all clinical severity spectrum.

A sensitivity analysis using inverse probability of treatment weighting (IPTW) in the initial cohort of 1375 patients was also performed to verify if a different propensity score method (IPTW vs propensity score matching) would lead to comparable results. Outcomes considered for this analysis were the primary outcome (functional independence at 90 days) and mortality within 90 days. The first was analysed using a weighted logistic regression model and the latter with a weighted Cox proportional hazard model, where weights were calculated using the estimated propensity score (PS) as 1/PS in those who were in the EVT ± IVT group and 1-1/PS in those in the IVT-only group. Weights were then normalised by their mean. Both models were also adjusted by the occlusion site, which was the other factor used for matching.

All tests were 2-tailed with a 5% level of significance and analyses were carried out with the SAS software (version 9.4, SAS Institute, Cary, NC, USA).

## Results

**Figure 1:**
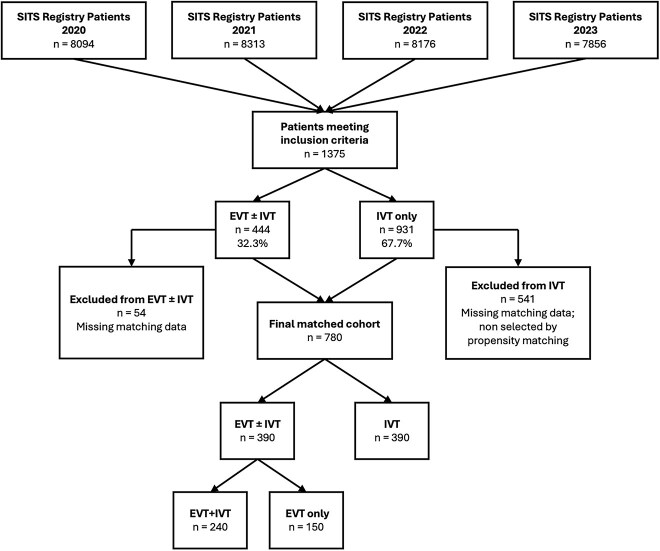
Patient selection flowchart for the SITS-MeVO study. Abbreviations: EVT = endovascular therapy; IVT = intravenous thrombolysis; SITS = Safe Implementation of Treatments in Stroke.

A total of 1375 patients with MeVO stroke met the inclusion criteria and were retrieved from the SITS database. Of these, 444 (32.3%) underwent EVT, while 931 (67.7%) received IVT alone. After excluding patients with missing data on primary outcome or matching variables (Figure 1), 780 patients were included in the final matched analysis, with 390 patients in each treatment group (EVT ± IVT vs IVT alone). In the EVT ± IVT group 150 patients received both treatments, while 240 received only mechanical thrombectomy. The patients excluded from the matching process (*n* = 595, 43.3%) differed significantly from the matched cohort (Table S1): they were slightly older (median age 79 vs 76 years, *P* < .0001), had lower baseline NIHSS scores (median 7 vs 10, *P* < .0001) and higher baseline disability (mRS 3–5; 9.75% vs 4.74%; *P* = .0005), and were predominantly treated with IVT alone (90.9% vs 50.0%).

### Baseline characteristics

The baseline characteristics were well-balanced between groups following propensity score matching (Table 1). The median age was 76 years (IQR 67–83), with no significant difference between the EVT ± IVT group (78 years; IQR 68–83) and the IVT-only group (75 years; IQR 67–83; *P* = .277). Male patients comprised approximately half of each group (49.7% vs 50.0%; *P* = .943). Most patients had favourable baseline functional status, with 95.3% having a pre-stroke mRS score of 0–2 (94.9% in EVT ± IVT group vs 95.6% in IVT group; *P* = .613).

**Table 1:** Population baseline characteristics.

**Characteristic**	**Total (*n* = 780)**	**EVT ± IVT (*n* = 390)**	**IVT (*n* = 390)**	
	***n* (%) or median (Q1–Q3)**	***n* (%) or median (Q1–Q3)**	***n* (%) or median (Q1–Q3)**	***P*-value**
**Demographics**				
** Age, years**	76 (67–83)	78 (68–83)	75 (67–83)	.277
** Sex, male**	389 (49.87)	194 (49.74)	195 (50.00)	.943
***Baseline mRS***				.613
** 0–2**	743 (95.26)	370 (94.87)	373 (95.64)	
** 3–5**	37 (4.74)	20 (5.13)	17 (4.36)	
**Risk factors**				
** Hypertension**	560 (71.79)	286 (73.33)	274 (70.26)	.340
** Diabetes mellitus**	131 (16.79)	68 (17.44)	63 (16.15)	.632
** Hyperlipidaemia**	296 (37.95)	151 (38.72)	145 (37.18)	.658
** Smoking, currently**	90 (12.33)	45 (12.26)	45 (12.40)	.954
** Atrial fibrillation**	204 (26.15)	114 (29.23)	90 (23.08)	.051
** Previous stroke**	71 (9.10)	37 (9.49)	34 (8.72)	.709
**Clinical presentation**				
** NIHSS Score on admission**	10 (6–15)	10 (6–15)	9 (5–15)	.271
**Treatment time metrics**				
** Median onset to needle time, min**	155 (118–215)	155 (118–210)^a^	155 (119–215)	.368
** Median onset to groyne time, min**	/	265 (200–385)	/	/
**Imaging**				
** Dense artery sign**	384 (49.23)	192 (49.23)	192 (49.23)	.966
***Occlusion site***				1.000
** M2**	690 (88.46)	345 (88.46)	345 (88.46)	
** M3 or more distal**	18 (2.31)	9 (2.31)	9 (2.31)	
** A1 or A2**	12 (1.54)	6 (1.54)	6 (1.54)	
** P1 or P2**	60 (7.69)	30 (7.69)	30 (7.69)	
***Occlusion side***				.560
** Right**	320 (41.03)	164 (42.05)	156 (40.00)	
** Left**	460 (58.97)	226 (57.95)	234 (60)	

^a^Data only applicable for patients who underwent EVT + IVT.

Abbreviations: EVT = endovascular therapy; IVT = intravenous thrombolysis.

Vascular risk factors were similarly distributed between groups, including hypertension (73.3% vs 70.3%; *P* = .340), diabetes mellitus (17.4% vs 16.2%; *P* = .632), current smoking habit (12.3% vs 12.4%; *P* = .954) and hyperlipidaemia (38.7% vs 37.2%; *P* = .658). Atrial fibrillation prevalence was numerically but not significantly higher in the EVT ± IVT group (29.2% vs 23.1%; *P* = .051).

Clinical presentation was comparable between groups, with median NIHSS scores of 10 (IQR 6–15) in the EVT ± IVT group and 9 (IQR 5–15) in the IVT-only group (*P* = .271). Median onset-to-needle time was similar between groups at 155 min (IQR 118–210 vs 119–215; *P* = .368). For patients receiving endovascular therapy, the median onset-to-groyne time was 265 min (IQR 200–385).

The distribution of occlusion sites was identical between groups due to matching criteria, with M2 segment occlusions representing the vast majority of cases (88.5%), followed by posterior circulation occlusions in P1 or P2 segments (7.7%). The distribution of left-sided vs right-sided occlusions was similar between groups (59.0% left-sided overall; *P* = .560).

### Primary outcome

The analysis of the primary outcome was performed after excluding 35 pairs of matched patients where at least 1 of the 2 was not functionally independent at baseline (mRS > 2), leaving a sample of 710 matched functionally independent (mRS 0–2) patients (355 per group). Functional independence at 90 days (mRS 0–2) was achieved in 60.6% of patients in the EVT ± IVT group compared to 60.9% in the IVT-only group (Table 2). This difference was not statistically significant (OR 0.99; 95% CI, 0.73–1.34; *P* = .939). The ordinal analysis including the entire sample of 780 patients confirmed no association between treatment and functional outcome at 90 days (adj. OR 0.88; 95% CI, 0.69–1.13; *P* = .327).

**Table 2:** Primary and secondary outcomes.

	**Total (*n* = 780)**	**EVT ± IVT (*n* = 390)**	**IVT (*n* = 390)**	
	***n* (%)**	***n* (%)**	***n* (%)**	***P*-value**
***Primary outcome***				
***mRS 0–2 at 90 days***	456 (58.46)	225 (57.69)	231 (59.23)	.663
***mRS 0–2 at 90 days in functionally independent (mRS > 2) at baseline***	431/710 (60.7)	215/355 (60.6)	216/355 (60.9)	.939
***Secondary outcomes***				
***In-hospital mortality*** ^***a***^	55 (7.07)	21 (5.40)	34 (8.74)	.069
***Successful recanalisation*** ^***b***^		179 (81.00)		
***TICI score*** ^***c***^				
** 0**		21 (9.50)		
** 1**		3 (1.36)		
** 2a**		18 (6.34)		
** 2b**		61 (25.00)		
** 3**		118 (53.39)		
***Revascularisation attempts*** ^***d***^				
** 1**		117 (59.39)		
** 2**		44 (22.34)		
** >3**		36 (18.29)		
***Ischaemic lesion size at 24 h CT*** ^***e***^				.415
** Total**	9 (3.50)	4 (2.99)	5 (4.07)	
** 2/3 of the vascular territory**	68 (26.46)	40 (29.85)	28 (22.76)	
** 1/3 of the vascular territory**	180 (70.04)	90 (67.16)	90 (73.17)	
***Haemorrhage at 24 h CT*** ^***f***^				**<.0001**
** None**	636 (85.14)	310 (81.58)	326 (88.83)	
** HI1**	43 (5.76)	31 (8.16)	12 (3.27)	
** HI2**	27 (3.61)	14 (3.68)	13 (3.54)	
** PH1**	27 (3.61)	15 (3.95)	12 (3.27)	
** PH2**	14 (1.87)	10 (2.63)	4 (1.09)	

^a^Defined as mRS 6 at discharge.

^b^Defined as TICI 2b or 3.

^c^Missing data from 192 subjects.

^d^Missing data from 221 subjects.

^e^According to SITS-MOST definition.

Abbreviations: EVT = endovascular therapy; IVT = intravenous thrombolysis; TICI = thrombolysis in cerebral infarction; HI1 = haemorrhagic infarction type 1; HI2 = haemorrhagic infarction type 2; PH1 = parenchymal haemorrhage type 1; PH2 = parenchymal haemorrhage type 2; SITS = Safe Implementation of Treatments in Stroke.

Among patients with pre-stroke mRS 0–1 (*n* = 671; 334 in EVT ± IVT, 337 in IVT), excellent functional outcome (mRS 0–1) at 90 days was achieved in 148 subjects of the EVT ± IVT group vs 171 subjects of the IVT group, respectively, 44.3% and 50.7% (*P* = .095).

### Secondary outcomes

Secondary outcome analysis results are listed in Table 2. In-hospital mortality occurred in 5.4% of patients in the EVT ± IVT group compared to 8.7% in the IVT-only group, showing a trend towards lower mortality with EVT, which approached statistical significance (*P* = .069).

The safety profile differed significantly between treatment groups, particularly regarding intracranial haemorrhage rates. While 11.2% of patients in the IVT-only group experienced an intracranial haemorrhage according to SITS-MOST classification, this proportion was higher in the EVT ± IVT group at 18.4% (*P* < .0001). As expected, contrast extravasation occurred exclusively in the EVT ± IVT group (5.3% vs 0%). However, the rates of sICH, all of which were parenchymal haemorrhage types 1 and 2 (PH1, PH2), were similar between the 2 groups, respectively, 6.6% in EVT ± IVT and 4.4% in IVT (*P* = .20; OR 1.54; 95% CI, 0.81–2.94; *P* = .20), indicating that the excess haemorrhagic risk was primarily limited to less severe forms.

Ischaemic lesion size on 24-h CT imaging, defined as proportion of total vascular territory, showed no significant difference between treatment groups (*P* = .415), with most patients in both groups demonstrating infarcts involving one-third or less of the vascular territory (67.2% in EVT ± IVT vs 73.2% in IVT-only).

Among patients who underwent endovascular procedures, data on TICI score and the number of revascularisation attempts were available in only 198/390 (50.8%) and 169/390 (43.3%) of cases, respectively. In the subset with available data, successful recanalisation (TICI 2b–3) was achieved in 81.0% of patients.

Given the predominance of M2 occlusions in our cohort (88.5%, *n* = 690), we performed a sensitivity analysis restricted to this anatomically homogeneous subgroup (Table S2). Among the 310 functionally independent at baseline matched pairs with M2 occlusions, functional independence rates at 90 days were nearly identical between treatment groups (62.6% in EVT ± IVT; 59.7% in IVT; *P* = .458). Analysis on secondary outcomes did not show any difference between the 2 groups either. These results confirm that the absence of functional benefit from EVT persists when analysis is restricted to the most common and anatomically uniform vessel occlusion in our study.

**Figure 2:**
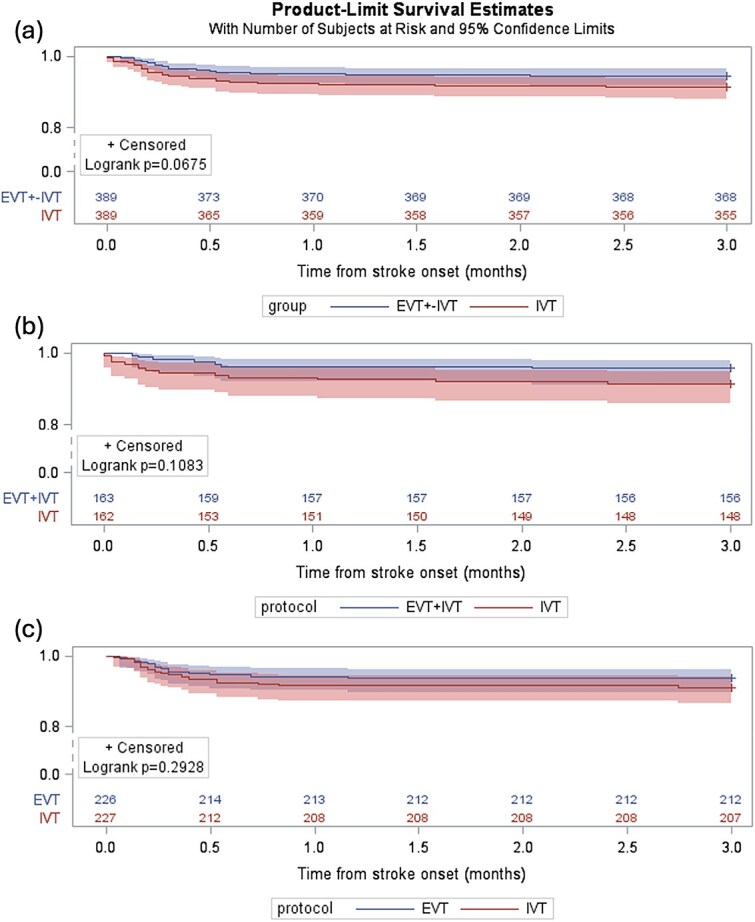
Kaplan–Meier survival curves (rescaled for clarity). From top to bottom, (a) EVT ± IVT vs IVT; (b) EVT + IVT vs IVT; (c) EVT vs IVT. Abbreviation: EVT = endovascular therapy; IVT = intravenous thrombolysis.

Kaplan–Meier survival analysis (Figure 2) demonstrated a trend towards improved cumulative survival rates with EVT ± IVT compared to IVT alone over the 3-month follow-up period (*P* = .068), consistent with the lower in-hospital mortality observed in the EVT group (5.4% vs 8.7%; *P* = .069). However, this survival benefit did not reach statistical significance and was not evident when analysing the EVT-only (*P* = .293) or EVT + IVT (*P* = .1083) subgroups separately, likely due to reduced statistical power in these subgroups.

### Stratified analysis

After excluding 35 functionally dependent pairs, interaction testing revealed no significant treatment-by-NIHSS interaction (*P* = .441) and a significant treatment-by-onset-to-treatment-time interaction (*P* = .007), indicating that the treatment effect did not vary significantly across severity strata, whereas it varied depending on timing. In the descriptive stratified analysis by NIHSS categories (Table S3), functional independence rates in the EVT ± IVT vs IVT-only groups were: 74.4% vs 83.1% for NIHSS < 5 (OR 0.59; 95% CI, 0.23–1.50; *P* = .267); 66.5% vs 63.9% for NIHSS 5–15 (OR 1.12; 95% CI, 0.75–1.68; *P* = .569) and 37.4% vs 34.5% for NIHSS > 15 (OR 1.13; 95% CI, 0.61–2.12; *P* = .697). When stratified by onset-to-treatment time (Table S3), functional independence rates were: 75.0% vs 58.2% for ≤ 180 min (OR 2.16; 95% CI, 1.06–4.38; *P* = .034); and 58.5% vs 67.9% for > 180 min (OR 0.67; 95% CI, 0.42–1.07; *P* = .094). These stratum-specific comparisons showed that when the onset-to-treatment time is below 180 min, the EVT ± IVT group had a higher probability of reaching a good functional outcome at 90 days.

### Sensitivity analysis

In the IPTW sensitivity analysis that included the entire original cohort, after excluding 25/444 functionally dependent patients in the EVT ± IVT and 78/931 in the IVT group, weighted logistic regression on the primary outcome showed no differences between the 2 groups (OR 0.85; 95% CI, 0.67–1.06; *P* = .144). The 90-day survival analysis with weighted Cox model showed that the risk of death was significantly lower in the EVT ± IVT compared to IVT (hazard ratio 0.50; 95% CI, 0.31–0.78; *P* = .0154).

## Discussion

This multicentre, real-world study based on the SITS registry from Italian centres, indicates that EVT, with or without IVT, does not provide superior functional outcomes compared to IVT alone in patients with MeVO stroke, even when excluding patients with a baseline mRS > 2. These findings align with the recently published results from the ESCAPE-MeVO and DISTAL randomised controlled trials, which similarly demonstrated no reduction in disability and mortality with thrombectomy compared to best medical treatment in this patient population.^9,10^

While our study employed a different methodological approach as a registry-based observational retrospective study with propensity score matching rather than randomised assignment, the convergence of results across different study designs strengthens the evidence base regarding the limited benefit of routine endovascular therapy for MeVO. The consistency of findings across randomised trials and registry data suggests that the absence of benefit is robust across different patient populations and clinical settings.^13–15^

While our findings align with ESCAPE-MeVO and DISTAL, a few differences in patient populations deserve explicit attention. Both trials enrolled patients with median baseline NIHSS of 7–8, compared to our matched cohort median of 10 and global cohort median of 8, suggesting that matched patients had moderately more severe strokes, while the whole cohort was more similar to the RCTs. The DISTAL trial reported approximately 69% IVT use and ESCAPE-MeVO approximately 61% in the medical management arm, whereas our IVT-only group had 100% IVT use by definition. Additionally, DISTAL excluded dominant M2 occlusions and include A1 or P1 segments, while ESCAPE-MeVO did include dominant M2 occlusions and excluded A1 or P1, whereas our cohort included all M2 occlusions regardless of dominance and encompassed A1/A2 and P1/P2 segments. These differences have important interpretive implications, since our cohort’s higher baseline severity and inclusion of more proximal occlusion sites would theoretically favour EVT, as more severe strokes with larger vessel occlusions might be expected to benefit more from mechanical recanalisation. Nevertheless, the fact that we still observed no functional benefit despite these characteristics makes our null finding particularly robust. The higher proportion of M2 occlusions in our cohort (68.4%) may partially reflect the inclusion of dominant M2 branches that would be classified as LVOs in other studies, though the comparable NIHSS scores between our groups suggest overall balance in stroke severity. A structured comparison between our study and RCTs is provided in Table 3, highlighting these population differences and their potential influence on generalisability.

**Table 3:** Comparison with randomised controlled trials ESCAPE-MeVO and DISTAL.

	**SITS-MeVO (*n* = 1375)**	**ESCAPE-MeVO (*n* = 529)**	**DISTAL (*n* = 543)**
**Characteristic**			
*** Median age, years***	78 (69–84)	75 (64–83)	77 (68–84)
*** Male sex, %***	48.0	53.7	56.0
*** Median baseline NIHSS, points***	8 (5–15)	8 (6–11)	6 (5–9)
*** Pre-stroke mRS 0–2, %***	93.2	Not specified	91.7
**Occlusion distribution**			
*** M2 occlusions, %***	68.4	43.5	44.0
*** M3 or more distal occlusions, %***	18.1	41.2	27.5
*** A1/A2/A3 occlusions, %***	2.3	4.6^a^	5.7
*** P1/P2/P3 occlusions, %***	11.1	10.7^a^	22.1
*** Dominant M2 included, %***	Unable to exclude	Not specified^b^	17.2
*** Tandem occlusions included, %***	Not included	Not included	6.1
**Treatment details**			
*** Numerosity in EVT arm, n***	444	255	271
*** Numerosity in control arm, n***	931	274	272
*** IVT use in EVT arm, %***	40.1	56.5	62.0
*** IVT use in control arm, %***	100 (by default)	60.5	68.8
*** Median onset-to-groyne, min***	130 (97–180)	95 (69–136)	87 (67–112)
**Outcomes**			
*** mRS 0–2 at 90 d (EVT), %***	56.9	51.7	56.5
*** mRS 0–2 at 90 d (control), %***	61.1	58.8	54.7
*** Symptomatic ICH (EVT), %***	6.7	5.4	14.3
*** Symptomatic ICH (control), %***	4.2	2.2	8.0

^a^ESCAPE-MeVO did not include A1 and P1 occlusions.

^b^ESCAPE-MeVO defined non-dominant M2 as M3 occlusions.

Abbreviations: EVT = endovascular therapy; IVT = intravenous thrombolysis; SITS = Safe Implementation of Treatments in Stroke.

Several methodological strengths enhance the reliability of these findings. The multicentre design across 82 Italian SITS centres provide substantial external validity and generalisability to routine clinical practice. The use of propensity score matching with careful attention to occlusion site matching helped minimise selection bias inherent in observational studies, creating comparable treatment groups despite the non-randomised design. The SITS registry infrastructure ensures standardised data collection protocols across participating centres, maintaining consistency in outcome definitions and follow-up procedures that strengthen the validity of cross-centre comparisons.

A particularly important finding of this study is the significantly higher rate of any intracranial haemorrhage in the EVT group (18.4% vs 11.2%; *P* < .0001), which was primarily driven by an increase in minor haemorrhagic events (HI1, HI2). While rates of more severe parenchymal haemorrhages (PH1, PH2) were comparable between groups, the clinical implications of these minor haemorrhages warrant consideration. Even small haemorrhagic transformation can have meaningful consequences including delays in mobilisation and rehabilitation initiation during the acute hospitalisation, complications in initiating or resuming anticoagulation therapy in patients with atrial fibrillation (approximately 25% of our cohort), potential prolongation of hospital length of stay requiring additional monitoring, and increased healthcare resource utilisation and costs. These complications may not directly affect 90-day mRS scores but represent a real burden of EVT that must be weighed against the absence of demonstrated functional benefit.

We observed a non-significant trend towards higher in-hospital mortality in the IVT-only group (8.7% vs 5.4%; *P* = .069). This finding should be regarded as hypothesis-generating rather than definitive, as several alternative explanations merit consideration beyond persistent vessel occlusion. First, patients undergoing EVT are by definition treated at comprehensive stroke centres with access to neurointerventional services, and these centres may provide more intensive monitoring and multidisciplinary care that could influence mortality independent of the revascularisation procedure itself. Second, residual confounding remains possible despite propensity score matching, particularly regarding unmeasured variables such as stroke mechanism or imaging characteristics not captured in the registry. Third, the finding appears somewhat inconsistent with the overall haemorrhagic transformation patterns, where EVT showed higher rates of haemorrhagic complications without a corresponding mortality increase. Finally, given that MeVO typically involves medium-to-distal occlusions where large territorial infarction with malignant oedema would be uncommon, alternative mechanisms would need to explain any mortality difference.

Interaction testing revealed a significant treatment-by-onset-to-treatment-time interaction (*P* = .007), where among patients treated within 180 min, EVT ± IVT was associated with a higher probability of good functional outcome at 90 days compared with IVT alone, while no benefit was observed beyond 180 min. This time-dependent effect could be explained by a higher efficacy of thrombectomy in the early therapeutic window, when collateral circulation may still sustain penumbral tissue before irreversible damage occurs. Importantly, this finding should be regarded as exploratory and hypothesis-generating, given the post hoc nature of the subgroup analysis and the limited sample size within the ≤ 180-min stratum, thus requiring confirmation in larger studies.

The M2-only sensitivity analysis further reinforces these conclusions by demonstrating that even within the most anatomically homogeneous and numerically dominant subgroup in our cohort, no functional benefit emerged from EVT, and, given that M2 occlusions represent the most common indication for EVT consideration in MeVO stroke, this specific finding has direct clinical relevance and supports the generalisability of our negative result to routine practice.

Taken together, these findings support a cautious approach to routine EVT in MeVO. While current evidence does not justify its widespread use, clinical judgement should be prioritised, and thrombectomy may still be considered in specific scenarios, such as outside the thrombolysis time window if the imaging profile (eg, high penumbra/core ratio) is favourable. As EVT device technology continues to evolve rapidly, the risk–benefit profile may change, warranting ongoing surveillance and re-evaluation of outcomes in future clinical studies.

The composition of our cohort provides important insights into routine care MeVO management patterns. Only 31.5% of eligible patients underwent EVT, with the remainder receiving IVT alone. This distribution likely reflects the gradual adoption of endovascular therapy for MeVOs during our study period (2020–2023), as well as the more conservative patient selection criteria historically applied to MeVO compared to LVO. The significant differences between matched and non-matched patients, particularly the age older than > 80 years, higher Rankin scale at baseline, and milder stroke severity in the excluded group, suggest that our results primarily apply to patients younger than 80 years old with moderate to severe MeVO strokes and better baseline functional status, who have traditionally been considered better candidates for EVT.

Our study has limitations to be considered. First, its retrospective nature introduces potential for unmeasured confounding, despite the use of propensity score matching. Second, the sample size, though comparable to other RCTs, was constrained by incomplete data in the original pre-matching cohort of 1375 patients.

Third, the matching process excluded 595 patients (43.3%) from the analysis. Only 444 (32.3%) had undergone EVT, reflecting the selective and evolving use of EVT for MeVO during the study period. Excluded patients were older, had milder strokes (lower NIHSS), higher baseline disability and were predominantly treated with IVT alone. This suggests a selection bias towards younger and/or more severely affected patients, limiting generalisability to elderly or mildly affected populations who are now increasingly considered for EVT.

Fourth, the classification of MeVO was limited by the anatomical definitions available in the SITS registry, which represents a significant constraint on interpretation. The registry does not distinguish between dominant and non-dominant M2 branches, and many authorities would classify dominant M2 occlusions as LVOs rather than MeVOs. This lack of differentiation may have led to the inclusion of some patients with what should be considered LVO, introducing heterogeneity into our cohort. Additionally, our inclusion of A1 and P1 segments, which some classifications consider large vessels,^16^ the exclusion of A3 and P3, often considered medium vessels, and the lumping together of M3 and more distal occlusions under a single category, further contribute to potential misclassification. While the comparable NIHSS scores between our matched groups (median 10 vs 9) suggest overall balance in stroke severity within the matched cohort, the broader anatomical heterogeneity remains a limitation when interpreting our findings and comparing them to studies with more refined vessel classifications.

Fifth, missing data affected the assessment of procedural outcomes: TICI scores and revascularisation attempts were available in only ~ 50% and 43% of EVT cases, respectively. Fifth, missing data affected the assessment of procedural outcomes: TICI scores and revascularisation attempts were available in only approximately 50% and 43% of EVT cases, respectively. Statistical analysis confirmed that this missingness pattern was not centre-dependent (chi-square test, *P* = .10), indicating random rather than systematic distribution. While random missingness reduces selection bias concerns, it can limit our ability to assess the relationship between recanalisation success and clinical outcomes. Moreover, unlike recent RCTs, our dataset included only revascularised patients, lacking a best medical treatment control group.

Lastly, infarct size thresholds were applied uniformly across vascular territories, although validation differs by territory,^17^ possibly introducing minor classification bias.

Additionally, our study spanned a period of evolving endovascular techniques and included the early COVID-19 pandemic, which likely impacted workflows and treatment decisions. These temporal and technical factors should be considered when interpreting outcome variability and the gradual adoption of EVT for MeVO.

Building on our findings and the alignment with recent RCTs, several clinical and research considerations emerge. The absence of functional benefit from EVT, combined with increased haemorrhagic risk, supports a conservative stance in future guideline updates. Endovascular mechanical thrombectomy should not be routinely recommended for MeVO stroke but considered selectively, especially when IVT is contraindicated or imaging suggests salvageable tissue.

Even among patients with moderate severity and good baseline status, who were more likely to receive EVT, no benefit emerged. This reinforces the need for refined selection algorithms integrating vessel anatomy, perfusion imaging and individualised risk–benefit assessment.

Given the resource intensity and lack of outcome improvement, EVT for MeVO may not be cost-effective at a population level. Targeted use in subgroups with clear potential for benefit is essential to justify procedural costs and avoid overtreatment.

The findings of this study suggest important opportunities for expanded investigation across the broader international SITS network: world-wide analysis could address several critical questions that remain unanswered, including the impact of healthcare system variations on treatment selection and outcomes, geographic differences in clinical practice patterns and the identification of specific patient subgroups who might derive benefit from endovascular therapy. Such an expanded dataset would provide substantially greater statistical power to detect clinically meaningful differences in rare but important outcomes and could enable more sophisticated analyses of treatment effect heterogeneity across different patient characteristics and clinical presentations.

## Conclusion

This multicentre, real-world registry study from 82 Italian centres demonstrates that endovascular therapy does not improve functional outcomes compared to IVT alone in patients with MeVO stroke, while being associated with higher rates of haemorrhagic complications. These findings, consistent with the recently published ESCAPE-MeVO and DISTAL randomised trials, provide robust real-world evidence against routine use of EVT for MeVO. Current evidence does not support routine endovascular therapy for MeVO stroke, therefore clinical decision-making should prioritise established therapies with proven benefit, particularly IVT, while carefully weighing the demonstrated risks of interventional approaches.

Future research should focus on identifying specific clinical and imaging characteristics that might predict treatment response in selected patient subgroups, potentially through international registry collaborations that can provide larger datasets with greater statistical power to detect clinically meaningful differences in patient subgroups. As endovascular device technology continues to evolve, ongoing surveillance through international registries and collaborative networks will be essential to detect changes in the risk–benefit profile that might eventually alter treatment recommendations for these complex cases.

## Supplementary Material

aakag020_Supplemental_materials

## Data Availability

The study protocol and additional supplementary information are available from the corresponding author upon reasonable request.

## List of SITS-MeVO Coinvestigators

Agostoni Elio, MD (Department of Neurology and Stroke Unit, ASST Grande Ospedale Metropolitano Niguarda, Milan, Italy), Baldi Antonio, MD (Stroke Unit, Ospedale Di Portogruaro, Portogruaro, Italy), Cardinali Patrizio, MD (Neurology Unit, AST Fermo, Marche, Italy), Cavallini Anna, MD (U.C. Malattie Cerebrovascolari e Stroke Unit, IRCCS Fondazione Mondino, Pavia, Italy), Ferrarese Carlo, MD (Department of Neurology, Fondazione IRCCS San Gerardo dei Tintori Monza, University of Milano Bicocca, Monza, Italy), Guidotti Mario, MD (Department of Neurology, Valduce Hospital, Como, Italy), Martusciello Gioacchino, MD (Neurology and Stroke Unit, AORN Sant'Anna e San Sebastiano, Caserta, Italy), Meineri Piero, MD (Department of Neurology, A.S.O. S. Croce e Carle, Cuneo, Italy), Napoletano Rosa, MD (UOSD Stroke Unit AOU San Giovanni Di Dio E Ruggi d'Aragona, Salerno, Italy), Pasquinucci Antonella, MD (Emergency Department, *S. Maria* Maddalena Hospital, Volterra, Italy), Plutino Andrea, MD (Stroke Unit, Marche Nord Riuniti Hospital, Fano, Italy), Reia Antonio, MD (Neurology Unit, PO S. Giuseppe Moscati, Aversa, Italy), Scaglione Gaspare, MD (Department of Neurology General Regional Hospital "F. Miulli" Acquaviva delle Fonti, Italy), Sicurella Luigi, MD (UOC Neurologia con Stroke Unit, ARNAS Garibaldi Catania), Tassi Rossana, MD (Stroke Unit, Urgency and Emergency Department, Azienda Ospedaliera Universitaria Senese, Siena, Italy)
