# Supplementary material for: Effectiveness and safety of endovascular therapy compared to intravenous thrombolysis in acute ischaemic stroke due to medium-vessel occlusions: a real-world multicentre study from the Italian SITS registry
Source: Eur Stroke J. 2026 Mar 29;11(3):aakag020. doi: 10.1093/esj/aakag020 (PMC13033145; doi:10.1093/esj/aakag020)
Supplement: aakag020_Supplemental_materials [file aakag020_supplemental_materials.docx]

***Figure SEQ Figure \* ARABIC 1. Kaplan-Meier 90-days survival curves.*** *From left-to-right, IVT versus EVT±IVT, IVT versus EVT+IVT, and IVT versus EVT. Log-rank expressed in each picture.*

| **Characteristic** | **Total (n = 1375)** | **Excluded (n = 595)** | **Included (n = 780)** |  |
| --- | --- | --- | --- | --- |
|  | **n (%) or median (Q1-Q3)** | **n (%) or median (Q1-Q3)** | **n (%) or median (Q1-Q3)** | **p-value** |
| **Demographics** |  |  |  |  |
| Age, years | 77 (68-84) | 79 (71-85) | 76 (67-83) | **< 0.0001** |
| Sex, male | 660 (48.0) | 271 (45.5) | 389 (49.9) | 0.112 |
| *Baseline mRS* |  |  |  | 0.003 |
| 0 - 2 | 1,272 (93.2) | 529 (90.4) | 743 (95.3) |  |
| 3 - 5 | 93 (6.8) | 56 (9.6) | 37 (4.7) |  |
| **Risk Factors** |  |  |  |  |
| Hypertension | 998 (72.7) | 438 (73.9) | 560 (71.8) | 0.395 |
| Diabetes Mellitus | 237 (17.2) | 106 (17.8) | 131 (16.8) | 0.620 |
| Hyperlipidemia | 519 (37.9) | 223 (38.0) | 296 (38.0) | 0.954 |
| Smoking, currently | 155 (12.2) | 65 (12.0) | 90 (12.3) | 0.978 |
| Atrial Fibrillation | 320 (23.3) | 116 (19.6) | 204 (26.2) | **0.004** |
| Previous Stroke | 134 (9.8) | 63 (10.8) | 71 (9.1) | 0.152 |
| **Clinical Presentation** |  |  |  |  |
| NIHSS Score on admission | 8 (5-15) | 7 (4-12) | 10 (6-15) | **< 0.0001** |
| **Treatment Time Metrics** |  |  |  |  |
| Median onset to needle time, minutes | 180 (135-270) | 165 (125-235) | 205 (145-300) | **< 0.0001** |
| Median onset to groin time, minutes | 155 (120-215) | 160 (120-225) | 155 (119-215) | 0.369 |
| **Imaging** |  |  |  |  |
| Dense artery sign | 544 (39.6) | 160 (26.9) | 384 (49.2) | **< 0.0001** |
| *Occlusion Site* |  |  |  | **< 0.0001** |
| M2 | 941 (68.4) | 251 (42.2) | 690 (88.5) |  |
| M3 or more distal | 249 (18.1) | 231 (38.8) | 18 (2.3) |  |
| A1 or A2 | 32 (2.3) | 20 (3.4) | 12 (1.5) |  |
| P1 or P2 | 153 (11.1) | 93 (15.6) | 60 (7.7) |  |
| *Occlusion Side* |  |  |  | 0.856 |
| Left | 808 (58.8) | 348 (58.5) | 460 (59.0) |  |
| Right | 567 (41.2) | 247 (41.5) | 320 (41.0) |  |
| **Treatment group** |  |  |  | **< 0.0001** |
| EVT ± IVT | 444 (32.3) | 54 (9.1) | 390 (50.0) |  |
| IVT only | 931 (67.7) | 541 (90.9) | 390 (50.0) |  |

## ***Table S1. Comparison of baseline characteristics between matched and excluded patients.***

## ***Abbreviations:****EVT, endovascular therapy; IVT, intravenous thrombolysis; mRS, modified Rankin Scale; NIHSS, National Institutes of Health Stroke Scale.*

|  | **EVT ± IVT (n = 345)** | **IVT (n = 345)** |  |
| --- | --- | --- | --- |
|  | **n (%)** | **n (%)** | **p-value** |
| ***Primary outcome*** |  |  |  |
| ***mRS 0-2 at 90 days*** | 204 (59.1) | 200 (58.0) | 0.817 |
| ***mRS 0-2 at 90 days in functionally independent (mRS>2) at baseline*** | 194/310 (62.6) | 185/310 (59.7) | 0.458 |
|  |  |  |  |
| ***mRS Distribution*** |  |  | 0.964 |
| mRS 0-2 | 204 (59.1) | 200 (58.0) |  |
| mRS 3-5 | 90 (26.1) | 93 (27.0) |  |
| mRS 6 | 51 (14.8) | 52 (15.1) |  |
|  |  |  |  |
| ***Secondary outcomes*** |  |  |  |
| ***In-hospital mortality^a^*** | 19 (5.5) | 32 (9.3) | 0.080 |
| ***Ischaemic lesion size at 24h CT^b^*** |  |  | 0.499 |
| Total | 4 (3.28) | 3 (3.48) |  |
| 2/3 of the vascular territory | 37 (30.33) | 27 (23.48) |  |
| 1/3 of the vascular territory | 81 (66.39) | 84 (73.04) |  |
| ***Haemorrhage at 24 h CT^c^*** |  |  | **< 0.0001** |
| None | 273 (81.25) | 287 (88.31) |  |
| HI1 | 28 (8.33) | 11 (3.38) |  |
| HI2 | 12 (3.57) | 12 (3.69) |  |
| PH1 | 13 (3.87) | 11 (3.38) |  |
| PH2 | 10 (2.98) | 4 (1.23) |  |

***Table S2. Sensitivity analysis on primary and secondary outcomes in M2 occlusions only.***

***Abbreviations:*** *EVT, endovascular therapy; IVT, intravenous thrombolysis; mRS, modified Rankin Scale; HI1, Haemorrhagic Infarction type 1; HI2, Haemorrhagic Infarction type 2; PH1, Parenchymal Haemorrhage type 1; PH2, Parenchymal Haemorrhage type 2.*

*^a^defined as mRS 6 at discharge; ^b^missing data from 286 subjects; ^c^according to SITS-MOST definition.*

| **Stratification Variable** | **EVT ± IVT (n = 355)** | **IVT (n = 355)** | **OR (95% CI)** | **p-value** |
| --- | --- | --- | --- | --- |
|  | **n/N (%)** | **n/N (%)** |  |  |
| **NIHSS Score** |  |  |  |  |
| < 5 | 29/39 (74.4) | 64/77 (83.1) | 0.59 (0.23-1.50) | 0.267 |
| 5-15 | 155/233 (66.5) | 122/191 (63.9) | 1.12 (0.75-1.68) | 0.569 |
| > 15 | 31/83 (37.4) | 30/87 (34.5) | 1.13 (0.61-2.12) | 0.697 |
| Interaction p-value |  |  |  | 0.441 |
| **Onset-to-Treatment Time** |  |  |  |  |
| ≤ 180 minutes | 36/48 (75.0) | 121/208 (58.2) | 2.16 (1.06-4.38) | **0.034** |
| > 180 minutes | 144/246 (58.5) | 76/112 (67.9) | 0.67 (0.42-1.07) | 0.094 |
| Interaction p-value |  |  |  | **0.007** |

***Table S3. Stratified Analysis of Primary Outcome (mRS 0-2 at 90 days).***

***Abbreviations:****EVT, endovascular therapy; IVT, intravenous thrombolysis; mRS, modified Rankin Scale; NIHSS, National Institutes of Health Stroke Scale; OR, odds ratio; CI, confidence interval.* ***Note:****Interaction p-values test whether treatment effects differ significantly between strata. Non-significant interactions indicate homogeneous treatment effects across subgroups.*
